# A Multistrategy Artificial Bee Colony Algorithm Enlightened by Variable Neighborhood Search

**DOI:** 10.1155/2019/2564754

**Published:** 2019-11-03

**Authors:** Wan-li Xiang, Yin-zhen Li, Rui-chun He, Xue-lei Meng, Mei-qing An

**Affiliations:** ^1^School of Traffic & Transportation, Lanzhou Jiaotong University, Lanzhou, Gansu 730070, China; ^2^Institute of Modern Logistics, Lanzhou Jiaotong University, Lanzhou, Gansu 730070, China

## Abstract

Artificial bee colony (ABC) has a good exploration ability against its exploitation ability. For enhancing its comprehensive performance, we proposed a multistrategy artificial bee colony (ABCVNS for short) based on the variable neighborhood search method. First, a search strategy candidate pool composed of two search strategies, i.e., ABC/best/1 and ABC/rand/1, is proposed and employed in the employed bee phase and onlooker bee phase. Second, we present another search strategy candidate pool which consists of the original random search strategy and the opposition-based learning method. Then, it is used to further balance the exploration and exploitation abilities in the scout bee phase. Last but not least, motivated by the scheme of neighborhood change of variable neighborhood search, a simple yet efficient choice mechanism of search strategies is presented. Subsequently, the effectiveness of ABCVNS is carried out on two test suites composed of fifty-eight problems. Furthermore, comparisons among ABCVNS and several famous methods are also carried out. The related experimental results clearly demonstrate the effectiveness and the superiority of ABCVNS.

## 1. Introduction

There are a vast lot of problems to be optimized in the social production and life. In order to achieve better solutions to these problems, a great number of algorithms have been developed. Owing to shortages (e.g., function's continuity needed) of deterministic approaches, various nature-enlightened algorithms had been developed to better deal with extremely difficult optimization problems. Generally speaking, during the past four decades, various researchers have developed different nature-inspired approaches such as genetic algorithms [1, 2], particle swarm optimization [3], differential evolution (called DE) [4], and artificial bee colony [5, 6].

Among them, artificial bee colony (ABC) is a distinguished representative of population-based global optimization methods, and it was first proposed by Karaboga in 2005 [5]. After that, comparative studies were carried out by Karaboga et al. [7, 8] among ABC, PSO, DE, GA, and so on. The comparative outcomes show its superiority when compared with those competitors. In addition, it needs fewer parameters. Afterwards, a great number of researchers show a lively interest in addressing ABC. Meanwhile, a lot of enhanced variants had been presented to solve various problems such as function optimization [9–22], vehicle routing problem [23], multiobjective optimization problems [24], and others [25–27].

Besides practical applications of ABC in the research fields listed above, many researchers concentrate on improving the performance of the traditional ABC to solve function optimization problems with different characteristics of nonconvexity, noncontinuity, separability, etc. For example, Alatas [9] proposed a chaotic ABC, in which both a chaotic initialization technique and a chaotic search method are proposed. Enlightened from the search process of particle swarm optimization, Zhu and Kwong [10] designed a novel search technique for improving ABC. In the proposed GABC, the search can effectively utilize the information of the global best individual. Motivated from the search strategy of DE [4], Gao and Liu [11] creatively developed a modified artificial bee colony (named MABC), in which a new equation named ABC/best/1 is proposed. To enhance the level of information sharing among various individuals, Akay and Karaboga [12] introduced a new control parameter called modification rate. It was used to randomly change parameters. Furthermore, it effectively enhances its exploitation ability. Frequently, the ABC was hybridized with other local approaches [13] to enhance its search ability. More recently, many researchers would like to utilize hybridization of multiple search strategies to balance the exploration and exploitation abilities of ABC [18, 21]. For instance, Gao et al. [18] first constructed a strategy candidate pool composed of three search strategies with different search abilities and then proposed an adaptive selection mechanism to select a search strategy for each individual based on previous search experiences. Moreover, the proposed algorithm, namely, MuABC, had achieved a better performance when compared with the standard ABC and other state-of-the-art algorithms. Kiran et al. [21] first chose five search strategies to form a strategy candidate pool and then designed a probabilistic selection scheme for choosing a search strategy during the evolving process. Furthermore, the proposed approach, called ABCVSS for short, outperformed the basic ABC, ABC variants, and other kinds of methods in terms of solution quality for most of the cases.

To better enhance the basic ABCs performance, a multistrategy ABC is proposed enlightened from the variable neighborhood search technique [28–30]. For convenience, it was named after ABCVNS, where both ABC/best/1 and ABC/rand/1 are used to construct the first search strategy candidate pool. It is utilized both in the first stage and in the second stage. Furthermore, the second search strategy candidate pool is employed in the scout bee phase, and it consists of an original random search strategy and an opposition-based learning method. Then, a novel mechanism of choice of search strategies is proposed inspired by the variable neighborhood search method. In addition, an opposition-based learning method is employed to initially generate a population with better diversities. To comprehensively show the advantage of ABCVNS, a few experiments on a large number of benchmark problems are conducted. Comparisons of ABCVNS and many other famous methods are also provided. The related comparative results demonstrate that the proposed ABCVNS can be regarded as a competitive method.

The remainder of the work is organized as follows.  Section 2 briefly describes the basic ABC. Next, a novel multistrategy ABC is proposed and described in detail in Section 3. In Section 4, a few comparative experiments are carried out and the comparative results are provided and discussed in detail. Finally, Section 5 concludes the work and puts forward a few future research directions.

## 2. Classical ABC

Enlightened from the collective intelligence behaviors of bee swarm [5], Karaboga developed ABC in 2005. In ABC, the bee swarm is divided into three groups. They are employed bees, onlooker bees, and scout bees, respectively. Among them, employed bees stand half of the swarm and onlooker bees form another half. As far as labor division of honey bees is concerned, the task of exploring nectar sources is undertaken by the employed bees. After that, they would pass the information of nectar amount onto onlooker bees. On the basis of the shared information, a food source is selected and exploited by an onlooker bee in turn with a certain percentage. If one employed bee or one onlooker bee exhausts a food source, then the corresponding bee would play a role of a scout bee, which will perform a random search to get out of a local trap.

Generally, by imitating the foraging behavior of artificial bee colony, the ABC is made up of four sequential phases. They are the initialization, employed bee, onlooker bee, and scout bee phases, respectively.

Before gathering the nectar of honey bees, a population of *N*_*p*_ artificial individuals is randomly generated according to the following equation to denote real-world honey bees:

(1) $$\begin{array}{c} x_{j}^{i}=x_{j}^{\min}+\left(x_{j}^{\max}-x_{j}^{\min}\right)\cdot \xi , \end{array}$$

where *i*=1,2,…, *N*_*p*_ and *j*=1,2,…, *D*; *x*_*j*_^max^ and *x*_*j*_^min^ represent the upper and lower bounds of the component *j*, respectively; and *ξ* is a random number in the range of [0,1). Randomly generated *D*-dimensional vector of *x*^*i*^ indicates an artificial agent. Meanwhile, we should predefine a suitable stopping criterion and the parameter *limit* employed for controlling appearances of scout bees.

Following the initialization phase, employed bees begin to explore food sources successively in the light of the following equation:

(2) $$\begin{array}{c} v_{j}=x_{j}^{i}+\varphi_{j}^{i}\cdot \left(x_{j}^{i}-x_{j}^{k}\right), \end{array}$$

where *i*=1,2,…, *N*_*p*_ and *k* ∈ {1,…, *N*_*p*_} together with *j* ∈ {1,…, *D*} are randomly produced by a uniform distribution. In addition, *k* has to be different from *i*. *φ*_*j*_^*i*^ is a randomly generated number between [−1,1].

Next, fitness values of artificial individuals are calculated as follows:

(3) $$\begin{array}{c} {\text{fit}}_{i}=\left\{\begin{array}{cc} \frac{1}{\left(1+f_{i}\right)},\; & \text{if }f_{i}\geq 0,\\ 1+\left|f_{i}\right|,\; & \text{otherwise,} \end{array}\right. \end{array}$$

where *f*_*i*_ and fit_*i*_ indicate the cost value and the fitness value of the *i*-th artificial individual, respectively.

At the beginning of the second stage, probability values are calculated according to the following formula:

(4) $$\begin{array}{c} p_{i}=\frac{{\text{fit}}_{i}}{\sum_{n=1}^{N_{p}}{\text{fit}}_{n}}, \end{array}$$

where *p*_*i*_ denotes the probability of the *i*-th artificial food source chosen by onlooker bees. It depends on nectar amounts of the corresponding food source. That is, the higher the fit_*i*_ is, the higher the chance of choosing the *i*-th food source is. In this context, employed bees pass information on to onlooker bees.

Based on equation (2), each onlooker bee chooses a food source in turn. Then, it begins to exploit around the corresponding food source.

Next, a predetermined parameter *limit* is employed to decide whether or not a scout bee occurs. Concretely speaking, provided that a bee continuously performs *limit* searches around the same food source, and it fails to achieve a better one, then the bee will become a scout bee. That is, it will randomly search for a new food source to jump out of a local trap in the light of the following equation:

(5) $$\begin{array}{c} v_{j}=x_{j}^{\min}+\left(x_{j}^{\max}-x_{j}^{\min}\right)\cdot \xi , \end{array}$$

where *j*=1,2,…, *D*. Besides, the other parameters are the same settings as those of equation (1).

During the foraging processes of the honey bee colony, they may cross some borders. That is, the artificial individuals/solutions may violate boundary constraints. To make solutions feasible, the following equation is employed to repair those infeasible solutions:

(6) $$\begin{array}{c} x_{j}^{i}=\left\{\begin{array}{cc} \frac{\left(x_{j}^{\min}+x_{j}^{\max}\right)}{2},\; & \text{if }x_{j}^{i}<x_{j}^{\min},\\ \frac{\left(x_{j}^{\min}+x_{j}^{\max}\right)}{2},\; & \text{if }x_{j}^{i}>x_{j}^{\max}. \end{array}\right. \end{array}$$

To summarize, after initializing a population, other stages of ABC are executed repeatedly until one halt condition is encountered.

## 3. A Multistrategy Artificial Bee Colony Algorithm

### 3.1. Initialize a Population in View of Opposition-Based Learning

First of all, a population of *N*_*p*_ artificial individuals is randomly produced according to equation (1). Based on the initial artificial individuals/solutions, some opposite solutions are generated to improve population diversities. That is, an opposition-based learning (OBL) method [31] is used to construct these opposite solutions. Since 2008, the OBL method has been widely applied in many population-based algorithms such as DE [32, 33] and ABC [11]. More concretely, equation (7) is employed here to produce oppositional vectors ${\overset{⌣}{x}}_{ij}$:

(7) $$\begin{array}{c} {\overset{⌣}{x}}_{j}^{i}=x_{j}^{\min}+x_{j}^{\max}-x_{j}^{i}, \end{array}$$

where *j*=1,2,…, *D*. The rest of the parameters are the same settings as those of equation (1).

By integrating the OBL and the random initialization approaches, the corresponding integrated initialization approach can be listed in Algorithm 1.

### 3.2. Search Strategy Candidate Pool

To simultaneously get better accuracies of solutions and a faster convergence performance, Gao et al. [18] and Kiran et al. [21] have proposed multiple search strategies to coordinate the exploitation and the exploration abilities of ABC from various perspectives, respectively. In addition, different neighbor search operators are also employed in other methods [34].

In this work, a new search strategy candidate pool is constructed with two search strategies. In addition, it is used in both the employed bee phase and the onlooker bee phase, respectively.

Enlightened from DE/best/1, Gao and Liu [11, 35, 36] first designed a very good equation named as ABC/best/1. In a few ABC variants like MABC [11], these enhanced versions achieved a better performance. This is the reason that the global best individual can guide faster the *i*-th individual around the best individual than a current individual *i*. Namely, ABC/best/1 is better than the traditional search strategy in the aspect of the exploitation ability. Therefore, ABC/best/1 is suitable to be integrated into the first candidate pool in this work. Its formula can be described as follows:

(8) $$\begin{array}{c} x_{j}^{i}=x_{j}^{\text{best}}+\left(2\;*\;\xi -1\right)\;*\;\left(x_{j}^{a}-x_{j}^{b}\right), \end{array}$$

where *j* ∈ {1,2,…, *D*}, *i* ∈ {1,2,…, *N*_*p*_}, and *a*, *b* ∈ {1,2,…, *N*_*p*_} are mutually different random integers. The two numbers *i* and best are also mutually different. best is the index of the global best individual in a population. *ξ* is a function to produce a uniformly distributed random number between [0, 1).

Enlightened from DE/rand/1, Gao and Liu [36] designed ABC/rand/1. Its exploitation ability is worse than that of ABC/best/1. However, its exploration ability is better than that of ABC/best/1. To better coordinate the two kinds of abilities, ABC/rand/1 is also added into the candidate pool. Its formula is described as follows:

(9) $$\begin{array}{c} x_{j}^{i}=x_{j}^{a}+\left(2\;*\;\xi -1\right)\;*\;\left(x_{j}^{b}-x_{j}^{c}\right), \end{array}$$

where *a*, *b*, *c* ∈ {1,2,…, *N*_*p*_} are randomly generated, and *a* ≠ *b* ≠ *c* ≠ *i*. The remainder of the parameters are the same settings as those of equation (8).

To further coordinate the exploration and the exploitation abilities of ABC, in the last stage, another search strategy candidate pool is constructed. The second candidate pool of search strategies is also composed of two search strategies.

Equation (5) is chosen as the first search strategy in the second strategy candidate pool. The second search strategy is described by equation (7). That is, the original random search strategy and the OBL search strategy are integrated to make up the second candidate pool of search strategies.

To improve the comprehensive performance of ABC, two candidate pools with different strategies are presented in this work.

### 3.3. The Choice of Search Strategies

As far as multiple search strategies are considered, the choice of search strategies also plays a key role during the process of improving the performance of ABC. Inspired by the variable neighborhood search (VNS) method [28–30], a simple yet efficient mechanism of choosing a search strategy for each bee is proposed.

In VNS, during the process of neighborhood changes, there are three steps: (i) set an iterative variable denoting a neighborhood as *k* = 1; (ii) if *k* is less than *k*_max_ predetermined, perform a local search in the *k*-th neighborhood, and then the objective value of new generated solution *x*′ is compared with that of the incumbent *x* previously achieved; and (iii) if an improvement is obtained, *k* is reset to its initial value and the incumbent is updated, namely, replace *x* with *x*′. Otherwise, the next neighborhood is considered, i.e., *k* = *k* + 1, and go to (ii). More details can be found in the literature [37].

In this work, a search strategy in a search strategy candidate pool is considered as a neighborhood. Thus, the main idea of choosing a search strategy (or changing neighborhood in VNS) is described as follows: (i) set *k* = 1; (ii) perform a search according to the search strategy *k* in the corresponding search strategy candidate pool by a bee; and (iii) if an improvement is obtained, the next bee continues to search a food source using the same search strategy. Otherwise, the next search strategy is chosen, namely, set *k* = *k* + 1, which also means that the next bee performs a search using the next search strategy. Provided that *k* is greater than its predefined maximum value, reset *k* to 1. Namely, let *k* = 1, which indicates that the first search strategy will be used by the next bee.

### 3.4. The Proposed Method

In the light of the aforementioned analysis, the major steps of ABCVNS are summarized in Algorithm 2.

## 4. Experimental Study and Discussion

### 4.1. Benchmark Problems and Experimental Settings

To test the effects of modifications of ABCVNS, twenty-eight benchmark problems given in Table 1 are employed in the following comparisons. Their detailed information can be found in the research work [21]. Many different kinds of optimization problems are covered by these benchmark problems, and more details can be found in [21].

Except for functions *f*_22_ and *f*_23_ in the experiments, all other benchmark problems are partitioned into two groups in the following experiments: One group consists of 30-dimensional functions, and another group consists of 60-dimensional functions. In the first group, functions *f*_22_ and *f*_23_ with *D* = 100 are tested. In the second group, functions *f*_22_ and *f*_23_ with *D* = 200 are tested. Accordingly, the number of maximum function evaluations (parameter maxFEs) is set to 15*e*4 and 30*e*4, respectively. To address the advantage of ABCVNS, it is compared with ABC at the beginning. As far as the two contenders are concerned, the other parameters of each algorithm are given below:

- ABC: the population size is 40, namely, *N*_*p*_ = 20 [18], and the control parameter limit is set to *N*_*p*_ *∗* *D* [8, 21]
- ABCVNS: the population size is 40, namely, *N*_*p*_ = 20 [18], and the control parameter limit is also set to *N*_*p*_ *∗* *D* [8, 21]

For the following experiments, the aforementioned values of parameters are used only if a change is mentioned. Moreover, the corresponding algorithm is independently run over 30 times while optimizing each benchmark problems.

### 4.2. Comparison of ABC vs. ABCVNS

**Figure 1:**
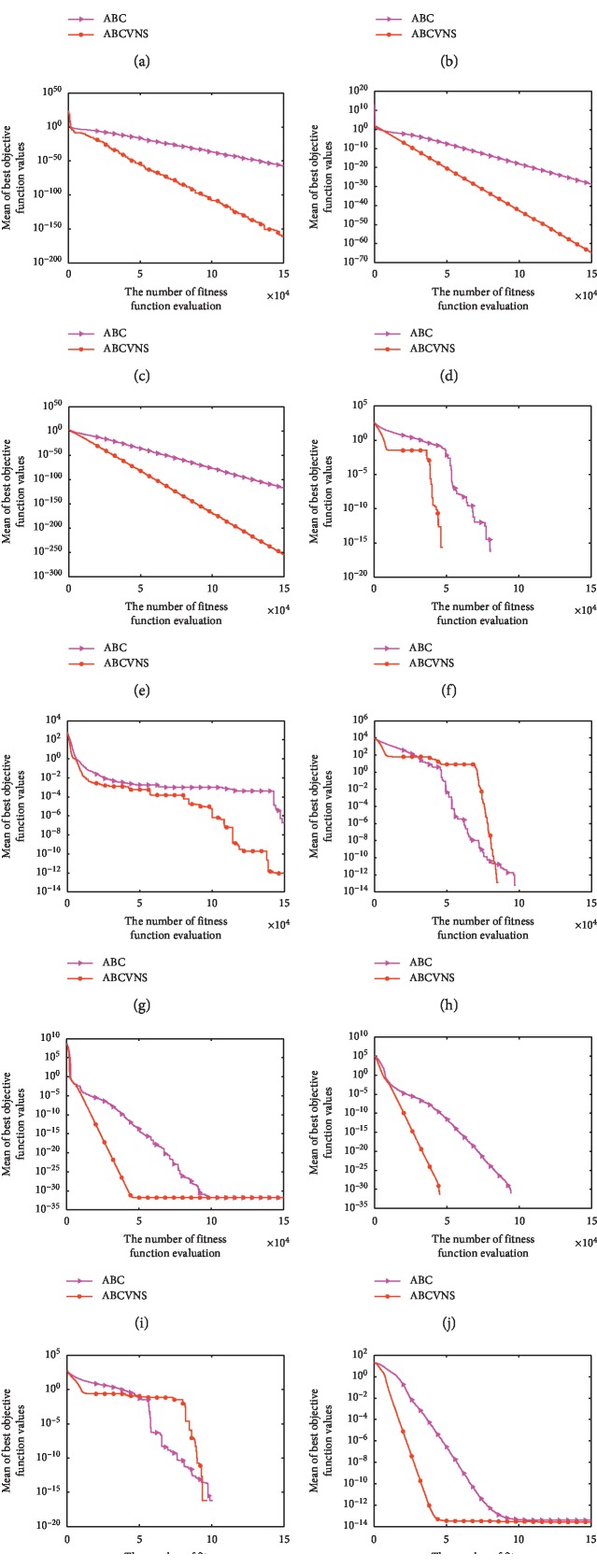
Convergence curves of ABCVNS and ABC on the 12 test functions at *D* = 30. (a) *f*_01_, (b) *f*_03_, (c) *f*_04_, (d) *f*_05_, (e) *f*_08_, (f) *f*_11_, (g) *f*_13_, (h) *f*_14_, (i) *f*_16_, (j) *f*_24_, (k) *f*_25_, and (l) *f*_27_.

**Figure 2:**
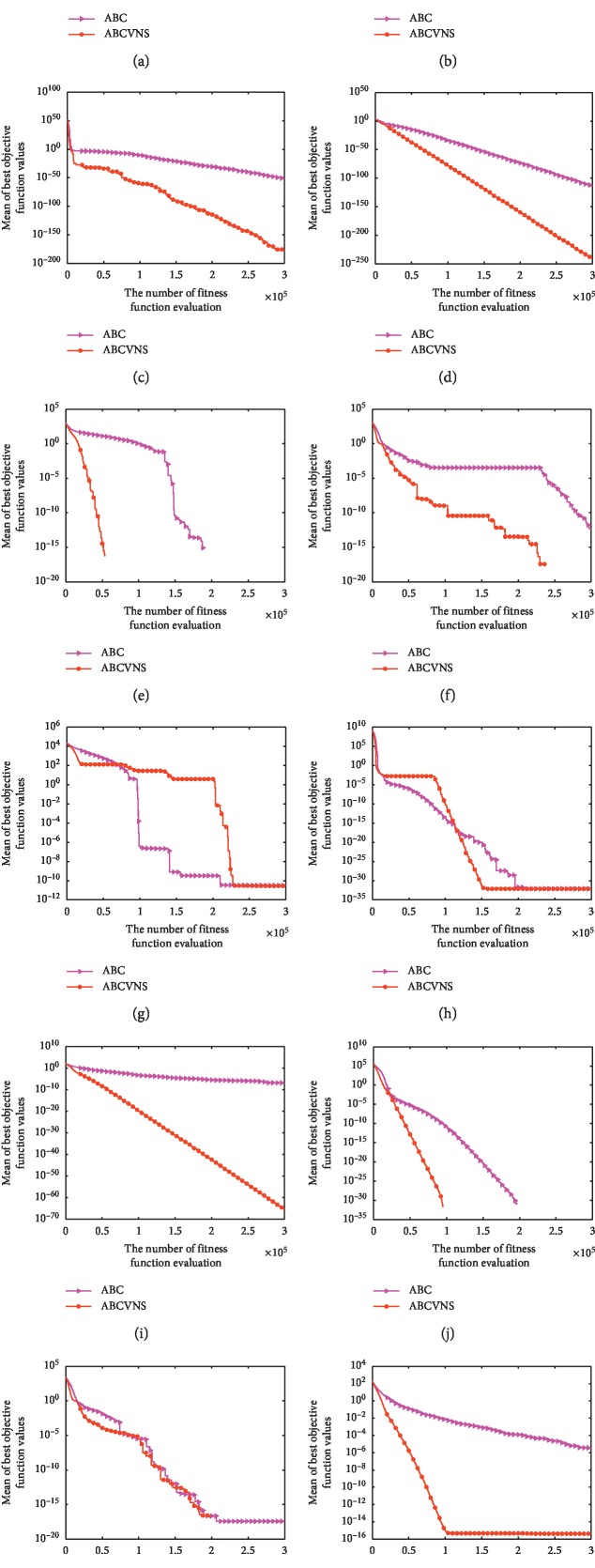
Convergence curves of ABCVNS and ABC on the 12 test functions at *D* = 60. (a) *f*_01_, (b) *f*_02_, (c) *f*_04_, (d) *f*_05_, (e) *f*_08_, (f) *f*_12_, (g) *f*_14_, (h) *f*_16_, (i) *f*_18_, (j) *f*_24_, (k) *f*_26_, and (l) *f*_28_.

To address the ABCVNSs effectiveness, comprehensive comparisons between ABCVNS and ABC are carried out. The sizes of test problems are set as *D* = 30 and *D* = 60, respectively. The corresponding results are provided in Tables 2 and 3. Concretely, we provide some statistical results such as standard deviation values (Std. listed in the ninth column of Tables 2 and 3) obtained by ABC and ABCVNS through 30 independent runs. Furthermore, the Wilcoxon signed rank tests between ABC and ABCVNS are also performed at the 5% significance level. The related significance statuses (Sig. for short) are listed in the tenth column of Tables 2 and 3, respectively. The symbols “+/≈/−” show that ABCVNS is better than, equal to, or inferior to ABC, respectively. Moreover, some representative convergence curves of ABC and ABCVNS are shown in Figures 1 and 2 to display the convergence rate of ABCVNS more clearly.

In the light of the tenth column of Table 2, ABCVNS is superior to or equal to ABC on almost all the test problems. In terms of mean values found by ABC and ABCVNS, the solution accuracies of ABCVNS are obviously enhanced with respect to those of ABC on nine benchmark functions, namely, *f*_01_, *f*_02_, *f*_03_, *f*_04_, *f*_05_, *f*_08_, *f*_18_, *f*_20_, and *f*_26_. As a note, the two algorithms, i.e., ABCVNS and ABC, are all coded in MATLAB R2014a, which implies that experimental results report zero until they are less than 1*e*−308, respectively. Furthermore, it is worth noting that ABCVNS achieves global optima on seven test problems, namely, *f*_07_, *f*_11_, *f*_12_, *f*_14_, *f*_20_, *f*_24_, and *f*_25_, whose characteristics include the characteristic of unimodal-separable (US) function, the characteristic of multimodal-separable (MS) function, and the characteristic of multimodal-nonseparable function. From the experimental results, it is clear that ABCVNS is obviously better than ABC.

As seen from Figure 1, ABCVNS is superior to ABC in terms of solution accuracy or convergence speed on most of the representative problems. Especially, the convergence speeds of ABCVNS are faster than those of ABC although the solution accuracies found by ABC are the same as those obtained by ABCVNS on some cases. The superiority of ABCVNS embodies on a few problems such as *f*_11_ and *f*_24_ is shown in Figures 1(f) and 1(j).

According to the last column of Table 3, we can find that ABCVNS is superior to or equal to ABC on almost all benchmark problems although the problem sizes raise from 30 to 60. These verify that ABCVNS is robust to problem sizes. Furthermore, ABCVNS achieves global optima on the seven benchmark functions, namely, *f*_07_, *f*_11_, *f*_12_, *f*_13_, *f*_20_, *f*_24_, and *f*_26_. Like the test problems with *D* = 30, the superiority of ABCVNS is also kept on the test problems with *D* = 60 in terms of solution accuracy as well as convergence speed as shown in Figure 2. Generally speaking, ABCVNS achieves better performance than ABC on most of the test problems. That is, our modifications on ABC are active.

### 4.3. Comparisons among ABCVNS as well as Other Famous ABCs

To further verify the superiority of ABCVNS, comparisons among ABCVNS together with a few well-known or recently published research works are done again. These famous contenders include GABC [10], ABCBest1 [35], MABC [11], and ABCVSS [21]. To make a fair comparison, the terminal condition for the approaches is the maximum number of function evaluations. It is set to 15e4 for 30-dimensional test problems (problems *f*_22_ and *f*_23_ with *D* = 100). It is set to 30*e*4 for 60-dimensional test problems (problems *f*_22_ and *f*_23_ with *D* = 200). The remaining parameters are the same as those employed in the research work [21]. As seen from Tables 4 and 5, some statistical results found by each algorithm are provided. For brevity, results of the competitors of ABCVNS are straightly adopted from the research findings of Kiran et al. [21].

Next, comparisons among ABCVNS and its competitors are carried out based on rank. The related results are listed in Tables 6 and 7, respectively. For the comparisons, mean value is first used to compare ABCVNS and its contender. If mean values found by ABCVNS and its contender are the same with each other, then standard deviation values are used to decide which method is better. If both of them are also the same with each other, then their ranks are the same.

As seen from Tables 4 and 6, ABCVNS is superior to or the same to its competitors like GABC, ABCBest1, and MABC together with ABCVSS in most of the cases. As seen from Table 6, ABCVNS gets the first prize in terms of the average rank among five algorithms. In sum, ABCVNS is a competitive algorithm.

From Tables 5 and 7, it can be observed that the best results are mainly achieved by ABCVNS or ABCVSS. Especially, ABCVNS still keeps original competitive advantage over its contenders although problem sizes increase from 30 to 60. As before, ABCVNS wins the first place compared with the four other famous approaches as shown in the last row of Table 7.

### 4.4. Comparison on CEC2014 Test Problems

To further address the superiority of ABCVNS, a comparison among ABCVNS, dABC [20], qABC [15], and ABCVSS [21] together with DFSABC_elite [38] is carried out on thirty CEC2014 benchmark problems [39] with *D* = 10.

For a fair comparison, the number of maximum function evaluations is employed as the halt condition. It is set to 10*e*4, which is also used in other research works [38, 39]. In addition, 25 runs are independently executed by each algorithm in the following experimental research studies. The statistical results are reported in Table 8. Note that the average and the standard deviation of the function error values *f*(*X*_best_) − *f*(*X*^*∗*^) are provided. Here, *X*_best_ denotes the best solution obtained by the corresponding algorithm in each run, and *X*^*∗*^ represents the real global optimal solution. Furthermore, except for results found by ABCVNS, all other reported ones are directly taken from the research work [38].

Both DFSABC_elite and ABCVNS are better than dABC, qABC, and ABCVSS as shown in Table 8. More concretely, DFSABC_elite wins the first place according to the average rank value reported in the last row of Table 8. Although ABCVNS is slightly inferior to DFSABC_elite, the orders of magnitude of mean values obtained by ABCVNS are very close to those searched by DFSABC_elite on ten test functions, i.e., F7, F10, F11, F12, F14, F16, F21, F22, F26, and F28. Especially, for the benchmark functions F26 and F28, the mean values of solutions found by ABCVNS are equal to those of solutions found by DFSABC_elite, respectively. DFSABC_elite wins ABCVNS on the two functions merely because the standard deviation values of solutions found by DFSABC_elite are slightly better than those of solutions obtained by ABCVNS. In sum, the proposed ABCVNS is also suitable for solving the very difficult problems.

To comprehensively investigate ABCVNS, another comparison among the three recent ABC variants and ABCVNS is carried out on twenty-two traditional complex functions with *D* = 30. These problems are also employed in the research work [38]. The experimental results are reported in Table 9. Among them, except for results found by ABCVNS, all other results are directly adopted from the research work [38].

As seen from Table 9, ABCVNS wins the first place according to the average rank value. Furthermore, it is worth noting that ABCVNS ranks 1st in thirteen of twenty-two functions. DFSABC_elite gets the second place. In addition, qABC is better than dABC. Especially, ABCVNS searches global optima on five test functions, i.e., *f*_07_, *f*_11_, *f*_12_, *f*_14_, and *f*_20_ with *D* = 30. Furthermore, the mean values of solutions found by ABCVNS are obviously better than those obtained by DFSABC_elite on six test functions, i.e., *f*_01_, *f*_02_, *f*_03_, *f*_04_, *f*_05_, and *f*_14_ with *D* = 30. However, the mean values of solutions found by DFSABC_elite are slightly superior or equal to those found by ABCVNS on the three test functions, i.e., *f*_09_, *f*_15_, and *f*_17_. To sum up, ABCVNS can be considered as a competitive method.

## 5. Conclusion

In this work, two search strategy candidate pools are proposed in order to overcome the contradiction between the fast convergence speed and the high solution accuracy. The first search strategy candidate pool is composed of two search strategies, i.e., ABC/best/1 and ABC/rand/1. The first search strategy candidate pool is employed in the employed bee phase and the onlooker bee phase. The second search strategy candidate pool also consists of two search strategies. They are the original random search strategy and the OBL method. In addition, it is employed in the scout bee phase to get a better compromise between the exploration ability and the exploitation ability. In addition, a simple yet efficient choice mechanism of search strategies is presented under the inspiration of variable neighborhood search algorithm. Then, a new variant of ABC is proposed, and it is called ABCVNS for short. To validate the convergence performance of the proposed ABCVNS, experiments on twenty-eight benchmark functions are performed. The basic comparison results between ABC and ABCVNS demonstrate that the modifications on ABC take effect. That is, ABCVNS obtains better performance than ABC. To fully validate the effectiveness of ABCVNS, it is compared with four other famous algorithms including ABCVSS. Related experimental results show that ABCVNS wins the first place according to the average rank. Subsequently, ABCVNS is further tested on a very difficulty test suite, i.e., CEC2014 benchmark functions. The related experimental results also demonstrate its superiority.

In a word, the proposed ABCVNS can be considered as a promising method. In the future, more smarter mechanisms of choosing different strategies is worth developing to take full advantage of various strategies.

## Figures and Tables

**Algorithm 1:**
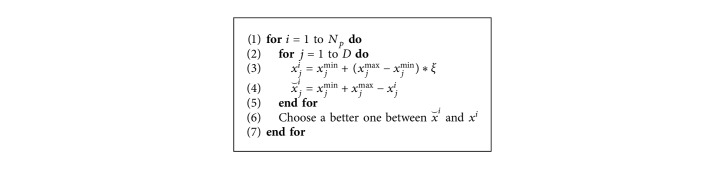
The integrated initialization method.

**Algorithm 2:**
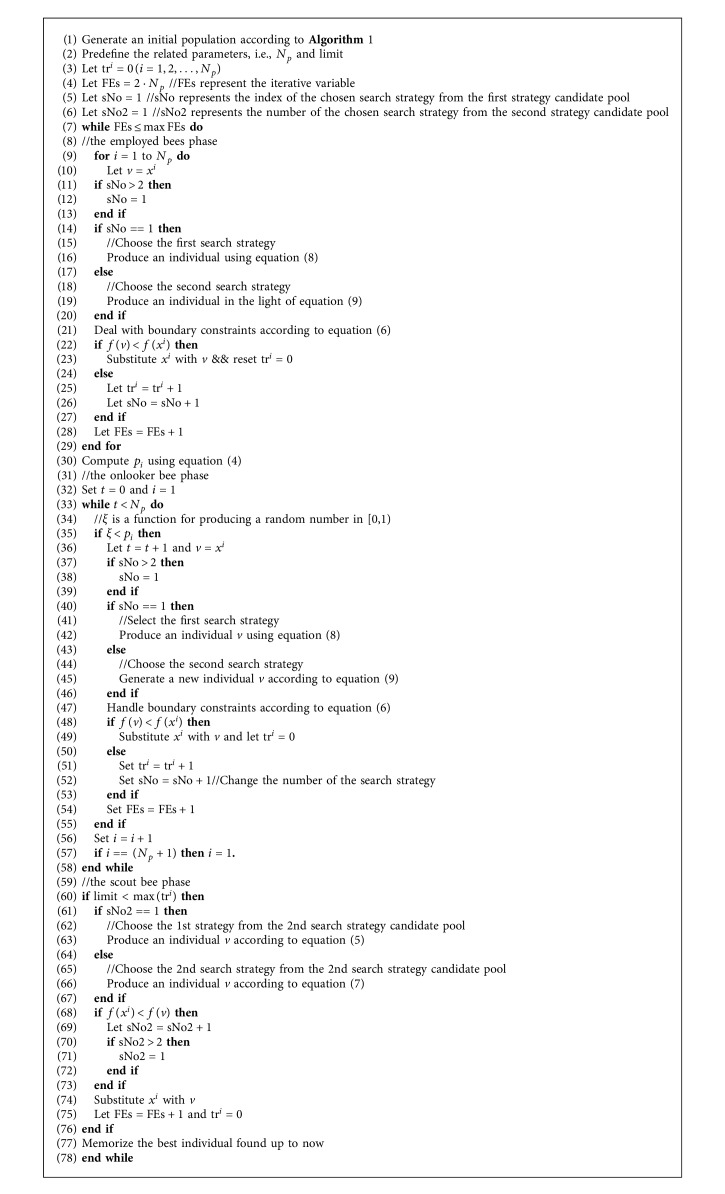
The framework of ABCVNS.

**Table 1:** Benchmark test problems.

Test problems	Domain range	Optimum
*f*_01_(*v*)=∑_*j*=1_^*D*^*v*_*j*_^2^	−100 ≤ *v*_*j*_ ≤ 100	0
*f*_02_(*v*)=∑_*j*=1_^*D*^(10^6^)^(*j* − 1)/(*n* − 1)^*v*_*j*_^2^	−100 ≤ *v*_*j*_ ≤ 100	0
*f*_03_(*v*)=∑_*j*=1_^*D*^*jv*_*j*_^2^	−10 ≤ *v*_*j*_ ≤ 10	0
*f*_04_(*v*)=∑_*j*=1_^*D*^|*v*_*j*_|^(*j*+1)^	−10 ≤ *v*_*j*_ ≤ 10	0
*f*_05_(*v*)=∑_*j*=1_^*D*^|*v*_*j*_|+∏_*j*=1_^*D*^|*v*_*j*_|	−10 ≤ *v*_*j*_ ≤ 10	0
*f*_06_(*v*)=mav_*j*_{|*v*_*j*_|, 1*≤j≤* *D*}	−100 ≤ *v*_*j*_ ≤ 100	0
*f*_07_(*v*)=∑_*j*=1_^*D*^(⌊*v*_*j*_+0.5⌋)^2^	−100 ≤ *v*_*j*_ ≤ 100	0
*f*_08_(*v*)=∑_*j*=1_^*D*^*jv*_*j*_^4^	−1.28 ≤ *v*_*j*_ ≤ 1.28	0
*f*_09_(*v*)=∑_*j*=1_^*D*^*jv*_*j*_^4^+random[0,1)	−1.28 ≤ *v*_*j*_ ≤ 1.28	0
*f*_10_(*v*)=∑_*j*=1_^*D*−1^[100(*v*_*j*+1_ − *v*_*j*_^2^)^2^+(*v*_*j*_ − 1)^2^]	−10 ≤ *v*_*j*_ ≤ 10	0
*f*_11_(*v*)=∑_*j*=1_^*D*^(*v*_*j*_^2^ − 10 cos(2*πv*_*j*_)+10)	−5.12 ≤ *v*_*j*_ ≤ 5.12	0
$\begin{array}{c} f_{12}\left(v\right)=\sum_{j=1}^{D}\left[y_{j}^{2}-10\;\cos\left(2\pi y_{j}\right)+10\right]\\ \text{where},y_{j}=\left\{\begin{array}{cc} v_{j,} & \text{if }\left|v_{j}\right|<0.5,\\ \text{round}\left(2v_{j}\right)/2, & \text{else }\left|v_{j}\right|\geq 0.5. \end{array}\right. \end{array}$	−5.12 ≤ *v*_*j*_ ≤ 5.12	0
$f_{13}\left(v\right)=\left(1/4000\right)\sum_{j=1}^{D}v_{j}^{2}-\prod_{j}^{D}\cos\left(v_{j}/\sqrt{j}\right)+1$	−600 ≤ *v*_*j*_ ≤ 600	0
$f_{14}\left(v\right)=-418.98288727243369\times D+\sum_{j=1}^{D}\left[-v_{j}\sin\left(\sqrt{\left|v_{j}\right|}\right)\right]$	−500 ≤ *v*_*j*_ ≤ 500	0
$f_{15}\left(v\right)=-20\text{evp}\left(-0.2\sqrt{\left(1/D\right)\sum_{j=1}^{D}v_{j}^{2}}\right)-\exp\left(\left(1/D\right)\sum_{j=1}^{D}\cos\left(2\pi v_{j}\right)\right)+20+e$	−32 ≤ *v*_*j*_ ≤ 32	0
$\begin{array}{c} f_{16}\left(v\right)=\frac{\pi }{D}\left\{10\;\sin^{2}\left(\pi y_{1}\right)+\sum_{j=1}^{D-1}{\left(y_{j}-1\right)}^{2}\right.\\ \left.\times \left[1+10\;\sin^{2}\left(\pi y_{j+1}\right)\right]{\left(y_{D}-1\right)}^{2}\right\}+\;\sum_{j=1}^{D}u\left(v_{j},10,100,4\right)\\ \text{where }y_{j}=1+\left(1/4\right)\left(v_{j}+1\right),u\left(v_{j},a,k,m\right)=\left\{\begin{array}{cc} k{\left(v_{j}-a\right)}^{m,} & v_{j}>a,\\ 0, & -a\leq v_{j}\leq a,\\ k{\left(-v_{j}-a\right)}^{m,} & v_{j}<-a. \end{array}\right. \end{array}$	−50 ≤ *v*_*j*_ ≤ 50	0
*f*_17_=0.1{sin^2^(3*πv*_1_)+∑_*j*=1_^*D*^(*v*_*j*_ − 1)^2^[1+ sin^2^(3*πv*_*j*_+1)]+(*v*_*n*_ − 1)^2^[1+ sin^2^(2*πv*_*n*_)]}+ ∑_*j*=1_^*D*^*u*(*v*_*j*_, 5,100,4)	−50 ≤ *v*_*j*_ ≤ 50	0
*f*_18_(*v*)=∑_*j*=1_^*D*^|*v*_*j*_sin(*v*_*j*_)+0.1*v*_*j*_|	−10 ≤ *v*_*j*_ ≤ 10	0
*f*_19_(*v*)=∑_*j*=1_^*D*−1^(*v*_*j*_ − 1)^2^[1+ sin^2^(3*πv*_*j*+1_)]+ sin^2^(3*πv*_1_)+|*v*_*D*_ − 1|[1+sjn^2^(3*πv*_*D*_)]	−10 ≤ *v*_*j*_ ≤ 10	0
*f*_20_(*v*)=∑_*j*=1_^*D*^(∑_*k*=0_^*k*_max_^[*a*^*k*^cos(2*πb*^*k*^(*v*_*j*_+0.5))]) − *D*∑_*k*=0_^*k*_mav_^[*a*^*k*^cos(2*πb*^*k*^0.5)], *a*=0.5, *b*=3, *k*_max_=20	0.5 ≤ *v*_*j*_ ≤ 0.5	0
$f_{21}\left(v\right)=0.5+\left(\sin^{2}\sqrt{\sum_{j=1}^{D}v_{j}^{2}}-0.5\right)/\left({\left(1+0.001\sum_{j=1}^{D}v_{j}^{2}\right)}^{2}\right)$	−100 ≤ *v*_*j*_ ≤ 100	0
*f*_22_(*v*)=(1/*D*)∑_*j*=1_^*D*^(*v*_*j*_^4^ − 16*v*_*j*_^2^+5*v*_*j*_)	−5 ≤ *v*_*j*_ ≤ 5	0
*f*_23_(*v*)=−∑_*j*=1_^*D*^sjn(*v*_*j*_)sin^20^(*jv*_*j*_^2^/*π*)	0 ≤ *v*_*j*_ ≤ *π*	0
*f*_24_(*v*)=∑_*j*=1_^*D*^*z*_*j*_^2^*z*=*v* − *o*	−100 ≤ *v*_*j*_ ≤ 100	0
*f*_25_(*v*)=∑_*j*=1_^*D*^(*z*_*j*_^2^ − 10 cos(2*πz*_*j*_)+10)*z*=*v* − *o*	−5.12 ≤ *v*_*j*_ ≤ 5.12	0
$\begin{array}{c} f_{26}\left(v\right)=\left(1/4000\right)\sum_{j=1}^{D}z_{j}^{2}-\prod_{j}^{D}\cos\left(z_{j}/\sqrt{j}\right)+1\\ z=v-o \end{array}$	−600 ≤ *v*_*j*_ ≤ 600*D*	0
$\begin{array}{c} f_{27}\left(v\right)=-20\text{evp}\left(-0.2\sqrt{\left(1/D\right)\sum_{j=1}^{D}z_{j}^{2}}\right)-\exp\left(\left(1/D\right)\sum_{j=1}^{D}\cos\left(2\pi z_{j}\right)\right)+20+e\\ z=v-o \end{array}$	−32 ≤ *v*_*j*_ ≤ 32	0
$\begin{array}{c} f_{28}\left(v\right)=\sum_{j=1}^{D}\sum_{j=1}^{D}\left|z_{j}\cdot \text{sjn}\left(z_{j}\right)+0.1\cdot z_{j}\right|\\ z=v-o \end{array}$	−10 ≤ *v*_*j*_ ≤ 10	0

**Table 2:** Objective values searched by ABC and ABCVNS for 30 and 100 (*f*_22_ and *f*_23_) test problems.

No.	Dim	maxFEs	Methods	Best	Worst	Median	Mean	Std.	Sig.
*f*_01_	30	15*e*4	ABC	4.76*e* − 057	1.25*e* − 053	1.67*e* − 055	9.15*e* − 055	2.37*e* − 054	†
			ABCVNS	7.57*e* − 139	1.81*e* − 123	1.69*e* − 128	7.26*e* − 125	3.30*e* − 124	
*f*_02_	30	15*e*4	ABC	6.66*e* − 048	1.15*e* − 044	2.82*e* − 046	1.43*e* − 045	2.67*e* − 045	†
			ABCVNS	3.22*e* − 137	7.26*e* − 121	5.32*e* − 126	2.77*e* − 122	1.33*e* − 121	
*f*_03_	30	15*e*4	ABC	4.82*e* − 059	2.24*e* − 054	4.02*e* − 057	8.23*e* − 056	4.09*e* − 055	†
			ABCVNS	9.03*e* − 136	9.87*e* − 125	1.39*e* − 130	3.33*e* − 126	1.80*e* − 125	
*f*_04_	30	15*e*4	ABC	1.84*e* − 067	1.71*e* − 056	1.45*e* − 061	6.48*e* − 058	3.12*e* − 057	†
			ABCVNS	0.00*e* + 00	1.43*e* − 160	7.01*e* − 216	4.76*e* − 162	2.61*e* − 161	
*f*_05_	30	15*e*4	ABC	4.42*e* − 030	4.88*e* − 029	1.50*e* − 029	1.80*e* − 029	1.13*e* − 029	†
			ABCVNS	5.58*e* − 071	1.66*e* − 064	1.72*e* − 067	1.38*e* − 065	3.56*e* − 065	
*f*_06_	30	15*e*4	ABC	2.78*e* − 001	1.55*e* + 000	6.44*e* − 001	7.15*e* − 001	3.04*e* − 001	†
			ABCVNS	4.61*e* − 003	1.58*e* − 002	1.06*e* − 002	1.03*e* − 002	2.88*e* − 003	
*f*_07_	30	15*e*4	ABC	0	0	0	0	0	≈
			ABCVNS	0	0	0	0	0	
*f*_08_	30	15*e*4	ABC	1.72*e* − 123	2.49*e* − 117	5.55*e* − 120	1.74*e* − 118	5.92*e* − 118	†
			ABCVNS	3.38*e* − 279	4.20*e* − 255	1.26*e* − 263	1.87*e* − 256	0	
*f*_09_	30	15*e*4	ABC	2.69*e* − 002	7.04*e* − 002	5.24*e* − 002	5.10*e* − 002	1.09*e* − 002	†
			ABCVNS	7.49*e* − 003	2.25*e* − 002	1.26*e* − 002	1.30*e* − 002	3.35*e* − 003	
*f*_10_	30	15*e*4	ABC	2.92*e* − 004	5.32*e* − 001	1.24*e* − 002	6.99*e* − 002	1.28*e* − 001	-
			ABCVNS	1.59*e* − 003	7.69*e* + 001	2.51*e* + 000	1.34*e* + 001	2.19*e* + 001	
*f*_11_	30	15*e*4	ABC	0	0	0	0	0	≈
			ABCVNS	0	0	0	0	0	
*f*_12_	30	15*e*4	ABC	0	0	0	0	0	≈
			ABCVNS	0	0	0	0	0	
*f*_13_	30	15*e*4	ABC	0	5.51*e* − 006	0	1.84*e* − 007	1.01*e* − 006	≈
			ABCVNS	0	2.68*e* − 011	0	8.96*e* − 013	4.89*e* − 012	
*f*_14_	30	15*e*4	ABC	0	0	0	0	0	≈
			ABCVNS	0	0	0	0	0	
*f*_15_	30	15*e*4	ABC	2.93*e* − 014	4.35*e* − 014	4.00*e* − 014	3.85*e* − 014	4.13*e* − 015	†
			ABCVNS	8.88*e* − 016	1.51*e* − 014	4.44*e* − 015	5.15*e* − 015	4.61*e* − 015	
*f*_16_	30	15*e*4	ABC	1.57*e* − 032	1.57*e* − 032	1.57*e* − 032	1.57*e* − 032	5.57*e* − 048	≈
			ABCVNS	1.57*e* − 032	1.57*e* − 032	1.57*e* − 032	1.57*e* − 032	5.57*e* − 048	
*f*_17_	30	15*e*4	ABC	1.35*e* − 032	1.35*e* − 032	1.35*e* − 032	1.35*e* − 032	5.57*e* − 048	≈
			ABCVNS	1.35*e* − 032	1.35*e* − 032	1.35*e* − 032	1.35*e* − 032	5.57*e* − 048	
*f*_18_	30	15*e*4	ABC	7.37*e* − 014	6.54*e* − 009	1.65*e* − 011	4.13*e* − 010	1.34*e* − 009	†
			ABCVNS	3.07*e* − 085	3.47*e* − 068	1.19*e* − 075	1.24*e* − 069	6.33*e* − 069	
*f*_19_	30	15*e*4	ABC	1.35*e* − 031	1.35*e* − 031	1.35*e* − 031	1.35*e* − 031	6.68*e* − 047	≈
			ABCVNS	1.35*e* − 031	1.35*e* − 031	1.35*e* − 031	1.35*e* − 031	6.68*e* − 047	
*f*_20_	30	15*e*4	ABC	0	7.11*e* − 015	0	7.11*e* − 016	2.17*e* − 015	≈
			ABCVNS	0	0	0	0	0	
*f*_21_	30	15*e*4	ABC	2.28*e* − 001	3.96*e* − 001	3.46*e* − 001	3.27*e* − 001	4.85*e* − 002	†
			ABCVNS	1.27*e* − 001	3.46*e* − 001	2.50*e* − 001	2.44*e* − 001	5.47*e* − 002	
*f*_22_	100	15*e*4	ABC	−78.33233	−78.33233	−78.33233	−78.33233	2.77*e* − 013	-
			ABCVNS	−78.33233	−78.33233	−78.33233	−78.33233	1.36*e* − 009	
*f*_23_	100	15*e*4	ABC	−97.03089	−95.93808	−96.36986	−96.40813		†
			ABCVNS	−9.96*e* + 001	−9.94*e* + 001	−9.95*e* + 001	−9.95*e* + 001	5.51*e* − 002	
*f*_24_	30	15*e*4	ABC	0	0	0	0	0	≈
			ABCVNS	0	0	0	0	0	
*f*_25_	30	15*e*4	ABC	0	0	0	0	0	≈
			ABCVNS	0	0	0	0	0	
*f*_26_	30	15*e*4	ABC	0	7.26*e* − 004	0	2.42*e* − 005	1.33*e* − 004	†
			ABCVNS	0	1.11*e* − 016	0	3.70*e* − 018	2.03*e* − 017	
*f*_27_	30	15*e*4	ABC	2.58*e* − 014	5.06*e* − 014	4.00*e* − 014	3.77*e* − 014	5.40*e* − 015	†
			ABCVNS	1.51*e* − 014	2.93*e* − 014	2.58*e* − 014	2.52*e* − 014	4.18*e* − 015	
*f*_28_	30	15*e*4	ABC	7.44*e* − 012	8.57*e* − 007	2.11*e* − 009	7.05*e* − 008	1.90*e* − 007	†
			ABCVNS	6.25*e* − 017	1.97*e* − 015	9.97*e* − 017	2.88*e* − 016	4.22*e* − 016	

“†” indicates ABCVNS is better than ABC by the Wilcoxon signed rank test at *α*=0.05.“-” means that ABCVNS is inferior to ABC. “≈” means that there is no significant difference between ABCVNS and ABC.

**Table 3:** Objective values searched by ABCVNS and ABC for 60 and 200 (*f*_22_ and *f*_23_) dimensional problems.

No.	Dim	maxFEs	Methods	Best	Worst	Median	Mean	Std.	Sig.
*f*_01_	60	30*e*4	ABC	2.28*e* − 055	1.03*e* − 052	4.89*e* − 054	1.35*e* − 053	2.21*e* − 053	†
			ABCVNS	9.82*e* − 129	1.24*e* − 119	2.49*e* − 123	9.44*e* − 121	2.92*e* − 120	
*f*_02_	60	30*e*4	ABC	1.61*e* − 046	5.23*e* − 043	7.51*e* − 045	4.08*e* − 044	1.01*e* − 043	†
			ABCVNS	1.62*e* − 124	1.52*e* − 114	4.00*e* − 118	9.47*e* − 116	3.01*e* − 115	
*f*_03_	60	30*e*4	ABC	5.28*e* − 057	4.01*e* − 054	3.04*e* − 055	6.76*e* − 055	9.21*e* − 055	†
			ABCVNS	2.77*e* − 128	1.43*e* − 118	3.45*e* − 123	7.26*e* − 120	2.69*e* − 119	
*f*_04_	60	30*e*4	ABC	4.25*e* − 060	5.99*e* − 050	9.36*e* − 056	2.27*e* − 051	1.09*e* − 050	†
			ABCVNS	.00*e* + 000	3.64*e* − 177	3.05*e* − 249	1.21*e* − 178	0.00*e* + 000	
*f*_05_	60	30*e*4	ABC	2.54*e* − 029	6.59*e* − 028	1.00*e* − 028	1.32*e* − 028	1.25*e* − 028	†
			ABCVNS	6.99*e* − 066	3.05*e* − 060	3.57*e* − 063	1.37*e* − 061	5.62*e* − 061	
*f*_06_	60	30*e*4	ABC	4.63*e* + 000	1.28*e* + 001	9.95*e* + 000	9.42*e* + 000	1.86*e* + 000	†
			ABCVNS	6.86*e* − 001	1.30*e* + 000	1.02*e* + 000	1.01*e* + 000	1.62*e* − 001	
*f*_07_	60	30*e*4	ABC	0	0	0	0	0	≈
			ABCVNS	0	0	0	0	0	
*f*_08_	60	30*e*4	ABC	5.91*e* − 119	7.61*e* − 114	1.25*e* − 115	8.84*e* − 115	1.90*e* − 114	†
			ABCVNS	2.69*e* − 258	1.52*e* − 239	5.16*e* − 251	5.07*e* − 241	0	
*f*_09_	60	30*e*4	ABC	6.29*e* − 002	1.23*e* − 001	1.03*e* − 001	1.00*e* − 001	1.61*e* − 002	†
			ABCVNS	2.43*e* − 002	3.91*e* − 002	3.08*e* − 002	3.11*e* − 002	4.60*e* − 003	
*f*_10_	60	30*e*4	ABC	1.11*e* − 003	7.30*e* − 001	3.65*e* − 002	7.67*e* − 002	1.41*e* − 001	-
			ABCVNS	1.69*e* − 002	1.43*e* + 002	7.23*e* + 001	5.79*e* + 001	3.71*e* + 001	
*f*_11_	60	30*e*4	ABC	0	0	0	0	0	≈
			ABCVNS	0	0	0	0	0	
*f*_12_	60	30*e*4	ABC	0	0	0	0	0	≈
			ABCVNS	0	0	0	0	0	
*f*_13_	60	30*e*4	ABC	0	1.27*e* − 011	0	4.24*e* − 013	2.32*e* − 012	≈
			ABCVNS	0	0	0	0	0	
*f*_14_	60	30*e*4	ABC	2.91*e* − 011	3.64*e* − 011	2.91*e* − 011	3.19*e* − 011	3.40*e* − 012	†
			ABCVNS	2.91*e* − 011	2.91*e* − 011	2.91*e* − 011	2.91*e* − 011	0	
*f*_15_	60	30*e*4	ABC	6.48*e* − 014	1.04*e* − 013	8.62*e* − 014	8.69*e* − 014	9.21*e* − 015	†
			ABCVNS	1.51*e* − 014	4.00*e* − 014	2.93*e* − 014	2.93*e* − 014	5.11*e* − 015	
*f*_16_	60	30*e*4	ABC	7.85*e* − 033	7.85*e* − 033	7.85*e* − 033	7.85*e* − 033	2.78*e* − 048	≈
			ABCVNS	7.85*e* − 033	7.85*e* − 033	7.85*e* − 033	7.85*e* − 033	2.78*e* − 048	
*f*_17_	60	30*e*4	ABC	1.35*e* − 032	1.35*e* − 032	1.35*e* − 032	1.35*e* − 032	5.57*e* − 048	≈
			ABCVNS	1.35*e* − 032	1.35*e* − 032	1.35*e* − 032	1.35*e* − 032	5.57*e* − 048	
*f*_18_	60	30*e*4	ABC	2.87*e* − 010	2.15*e* − 006	6.63*e* − 009	1.60*e* − 007	4.48*e* − 007	†
			ABCVNS	2.23*e* − 080	4.22*e* − 065	2.40*e* − 067	3.78*e* − 066	9.53*e* − 066	
*f*_19_	60	30*e*4	ABC	1.35*e* − 031	1.35*e* − 031	1.35*e* − 031	1.35*e* − 031	6.68*e* − 047	≈
			ABCVNS	1.35*e* − 031	1.35*e* − 031	1.35*e* − 031	1.35*e* − 031	6.68*e* − 047	
*f*_20_	60	30*e*4	ABC	0	2.84*e* − 014	1.42*e* − 014	1.47*e* − 014	9.50*e* − 015	†
			ABCVNS	0	0	0	0	0	
*f*_21_	60	30*e*4	ABC	4.30*e* − 001	4.89*e* − 001	4.80*e* − 001	4.76*e* − 001	1.21*e* − 002	†
			ABCVNS	3.46*e* − 001	4.72*e* − 001	4.52*e* − 001	4.44*e* − 001	2.83*e* − 002	
*f*_22_	200	30*e*4	ABC	−78.33233	−78.33233	−78.33233	−78.33233	6.27*e* − 013	†
			ABCVNS	−78.33233	−78.33233	−78.33233	−78.33233	6.67*e* − 014	
*f*_23_	200	30*e*4	ABC	−192.8520	−191.0752	−192.0762	−192.0878	4.09*e* − 001	†
			ABCVNS	−1.99*e* + 002	−1.99*e* + 002	−1.99*e* + 002	−1.99*e* + 002	7.25*e* − 002	
*f*_24_	60	30*e*4	ABC	0	0	0	0	0	≈
			ABCVNS	0	0	0	0	0	
*f*_25_	60	30*e*4	ABC	0	0	0	0	0	≈
			ABCVNS	0	0.0823	0	0.0027	0.0150	
*f*_26_	60	30*e*4	ABC	0	1.11*e* − 016	0	3.70*e* − 018	2.03*e* − 017	≈
			ABCVNS	0	0	0	0	0	
*f*_27_	60	30*e*4	ABC	7.55*e* − 014	9.33*e* − 014	8.62*e* − 014	8.49*e* − 014	6.56*e* − 015	†
			ABCVNS	5.06*e* − 014	7.55*e* − 014	6.48*e* − 014	6.19*e* − 014	6.26*e* − 015	
*f*_28_	60	30*e*4	ABC	2.01*e* − 009	2.80*e* − 005	3.86*e* − 007	3.37*e* − 006	6.57*e* − 006	†
			ABCVNS	1.56*e* − 016	2.06*e* − 015	2.17*e* − 016	4.13*e* − 016	4.21*e* − 016	

“†” indicates ABCVNS is superior to ABC by the Wilcoxon signed rank test at *α*=0.05. “-” means that ABCVNS is inferior to ABC. “≈” means that there is no significant difference between ABC and ABCVNS.

**Table 4:** Comparisons of ABCVNS and other ABCs over 30 independent runs on the 30 and 100 (*f*_22_ and *f*_23_) dimensional problems.

No.	maxFEs	GABC	ABCBest1	MABC	ABCVSS	ABCVNS
*f*_01_	15*e*4	4.62*e* − 16 (7.12*e* − 17)	3.11*e* − 47 (3.44*e* − 47)	9.43*e* − 32 (6.67*e* − 32)	1.53*e* − 081 (8.37*e* − 081)	**7.26e − 125 (3.30e − 124)**
*f*_02_	15*e*4	3.62*e* − 16 (7.88*e* − 17)	5.35*e* − 44 (4.91*e* − 44)	3.66*e* − 28 (5.96*e* − 28)	4.82*e* − 082 (2.63*e* − 081)	**2.77e − 122 (1.33e − 121)**
*f*_03_	15*e*4	4.55*e* − 16 (7.00*e* − 17)	6.50*e* − 48 (6.04*e* − 48)	2.10*e* − 32 (1.56*e* − 32)	3.19*e* − 089 (1.48*e* − 088)	**3.33e − 126 (1.80e − 125)**
*f*_04_	15*e*4	1.64*e* − 17 (8.07*e* − 18)	1.77*e* − 86 (7.02*e* − 86)	2.70*e* − 69 (5.38*e* − 69)	5.55*e* − 115 (3.04*e* − 114)	**4.76e − 162 (2.61e − 161)**
*f*_05_	15*e*4	1.35*e* − 15 (1.36*e* − 16)	2.10*e* − 25 (9.08*e* − 26)	2.40*e* − 17 (9.02*e* − 18)	7.89*e* − 043 (4.32*e* − 042)	**1.38e − 065 (3.56e − 065)**
*f*_06_	15*e*4	2.18*e* − 01 (4.01*e* − 02)	2.18*e* + 00 (3.27*e* − 01)	1.02*e* + 01 (1.49*e* + 00)	4.08*e* − 002 (2.20*e* − 002)	**1.03e − 002 (2.88e − 003)**
*f*_07_	15*e*4	0 (0)	0 (0)	0 (0)	0 (0)	**0 (0)**
*f*_08_	15*e*4	1.21*e* − 16 (3.99*e* − 17)	2.63*e* − 97 (3.75*e* − 97)	1.45*e* − 67 (2.28*e* − 67)	3.25*e* − 154 (1.78*e* − 153)	**1.87e − 256 (0.00e + 000)**
*f*_09_	15*e*4	2.03*e* − 02 (5.74*e* − 03)	2.06*e* − 02 (4.75*e* − 03)	3.71*e* − 02 (8.53*e* − 03)	1.81*e* − 002 (5.27*e* − 003)	**1.30e − 002 (3.35e − 003)**
*f*_10_	15*e*4	**3.21e − 01 (8.21e − 01)**	1.49*e* + 01 (2.87*e* + 01)	6.11*e* − 01 (4.55*e* − 01)	3.87*e* − 001 (1.54*e* + 000)	**1.34e + 001 (2.19e + 001)**
*f*_11_	15*e*4	0 (0)	0 (0)	0 (0)	0 (0)	**0 (0)**
*f*_12_	15*e*4	0 (0)	0 (0)	0 (0)	0 (0)	**0 (0)**
*f*_13_	15*e*4	3.70*e* − 17 (5.32*e* − 17)	0 (0)	0 (0)	**0 (0)**	**8.96e − 013 (4.89e − 012)**
*f*_14_	15*e*4	9.42*e* − 02 (5.16*e* − 01)	1.33*e* − 12 (8.18*e* − 13)	1.21*e* − 13 (4.53*e* − 13)	4.85*e* − 013 (8.18*e* − 013)	**0 (0)**
*f*_15_	15*e*4	3.20*e* − 14 (3.36*e* − 15)	3.01*e* − 14 (2.91*e* − 15)	4.13*e* − 14 (2.17*e* − 15)	2.45*e* − 014 (4.00*e* − 015)	**5.15e − 015 (4.61e − 015)**
*f*_16_	15*e*4	4.12*e* − 16 (8.36*e* − 17)	1.57*e* − 32 (5.57*e* − 48)	1.90*e* − 32 (3.70*e* − 33)	1.57*e* − 032 (5.57*e* − 048)	**1.57e − 032 (5.57e − 048)**
*f*_17_	15*e*4	4.01*e* − 16 (8.19*e* − 17)	1.35*e* − 32 (5.57*e* − 48)	2.23*e* − 31 (1.46*e* − 31)	1.35*e* − 032 (5.57*e* − 048)	**1.35e − 032 (5.57e − 048)**
*f*_18_	15*e*4	3.41*e* − 09 (1.13*e* − 08)	3.00*e* − 16 (8.99*e* − 16)	1.58*e* − 16 (2.48*e* − 16)	3.66*e* − 044 (1.93*e* − 043)	**1.24e − 069 (6.33e − 069)**
*f*_19_	15*e*4	3.28*e* − 16 (5.03*e* − 17)	1.35*e* − 31 (6.68*e* − 47)	1.48*e* − 31 (2.30*e* − 32)	1.35*e* − 031 (6.68*e* − 047)	**1.35e − 031 (6.68e − 047)**
*f*_20_	15*e*4	0 (0)	4.74*e* − 16 (1.80*e* − 15)	0 (0)	0 (0)	**0 (0)**
*f*_21_	15*e*4	2.66*e* − 01 (4.39*e* − 02)	**2.39e − 01 (6.13e − 02)**	2.95*e* − 01 (3.17*e* − 02)	2.84*e* − 001 (5.69*e* − 002)	**2.44e − 001 (5.47e − 002)**
*f*_22_	15*e*4	**−7.83e + 01 (2.94e − 14)**	−7.83*e* + 01 (6.68*e* − 12)	−7.83*e* + 01 (2.06*e* − 07)	−7.83*e* + 01 (3.02*e* − 010)	**−7.83e + 01 (1.36e − 009)**
*f*_23_	15*e*4	−9.94*e* + 01 (4.18*e* − 02)	−9.57*e* + 01 (3.89*e* − 01)	−9.07*e* + 01 (5.03*e* − 01)	−9.94*e* + 01 (8.84*e* − 002)	**−9.95e + 001 (5.51e − 002)**
*f*_24_	15*e*4	4.38*e* − 16 (8.43*e* − 17)	0 (0)	0 (0)	0 (0)	**0 (0)**
*f*_25_	15*e*4	0 (0)	0 (0)	0 (0)	0 (0)	**0 (0)**
*f*_26_	15*e*4	3.33*e* − 17 (5.17*e* − 17)	8.81*e* − 16 (3.38*e* − 15)	0 (0)	**0 (0)**	**3.70e − 018 (2.03e − 017)**
*f*_27_	15*e*4	3.20*e* − 14 (2.80*e* − 15)	2.89*e* − 14 (2.59*e* − 15)	4.92*e* − 14 (5.31*e* − 15)	2.53*e* − 014 (4.55*e* − 015)	**2.52e − 014 (4.18e − 015)**
*f*_28_	15*e*4	6.65*e* − 08 (2.39*e* − 07)	1.50*e* − 16 (2.48*e* − 16)	1.38*e* − 16 (8.11*e* − 17)	**7.49e − 017 (1.48e − 017)**	**2.88e − 016 (4.22e − 016)**

Bold entity refers to one of the best results.

**Table 5:** Comparison of the ranks of the algorithms for the results of 30 and 100 (*f*_22_ and *f*_23_) dimensional problems.

No.	Index	GABC	ABCBest1	MABC	ABCVSS	ABCVNS
*f*_01_	Rank	5	3	4	2	1
*f*_02_	Rank	5	3	4	2	1
*f*_03_	Rank	5	3	4	2	1
*f*_04_	Rank	5	3	4	2	1
*f*_05_	Rank	5	3	4	2	1
*f*_06_	Rank	3	4	5	2	1
*f*_07_	Rank	1	1	1	1	1
*f*_08_	Rank	5	3	4	2	1
*f*_09_	Rank	3	4	5	2	1
*f*_10_	Rank	1	5	3	2	4
*f*_11_	Rank	1	1	1	1	1
*f*_12_	Rank	1	1	1	1	1
*f*_13_	Rank	4	1	1	1	5
*f*_14_	Rank	5	4	2	3	1
*f*_15_	Rank	4	3	5	2	1
*f*_16_	Rank	5	1	4	1	1
*f*_17_	Rank	5	1	4	1	1
*f*_18_	Rank	5	4	3	2	1
*f*_19_	Rank	5	1	4	1	1
*f*_20_	Rank	1	5	1	1	1
*f*_21_	Rank	3	1	5	4	2
*f*_22_	Rank	1	2	5	3	4
*f*_23_	Rank	2	4	5	3	1
*f*_24_	Rank	5	1	1	1	1
*f*_25_	Rank	1	1	1	1	1
*f*_26_	Rank	4	5	1	1	3
*f*_27_	Rank	4	3	5	2	1
*f*_28_	Rank	5	3	2	1	4

Average rank		3.536	2.643	3.179	1.750	1.571
Final rank		5	3	4	2	1

**Table 6:** Comparison of ABCVNS and other ABCs over 30 independent runs on the 60 and 200 (*f*_22_ and *f*_23_) dimensional problems.

No.	maxFEs	GABC	ABCBest1	MABC	ABCVSS	ABCVNS
*f*_01_	30*e*4	1.06*e* − 15 (1.21*e* − 16)	3.92*e* − 44 (2.64*e* − 44)	6.03*e* − 29 (4.31*e* − 29)	1.09*e* − 083 (5.01*e* − 083)	**9.44e − 121 (2.92e − 120)**
*f*_02_	30*e*4	8.97*e* − 16 (9.29*e* − 17)	1.70*e* − 41 (9.16*e* − 42)	3.51*e* − 25 (2.72*e* − 25)	1.01*e* − 082 (5.54*e* − 082)	**9.47e − 116 (3.01e − 115)**
*f*_03_	30*e*4	1.04*e* − 15 (1.27*e* − 16)	2.06*e* − 44 (1.83*e* − 44)	1.39*e* − 29 (8.84*e* − 30)	8.17*e* − 086 (4.47*e* − 085)	**7.26e − 120 (2.69e − 119)**
*f*_04_	30*e*4	2.85*e* − 17 (1.01*e* − 17)	8.74*e* − 74 (4.63*e* − 73)	3.00*e* − 62 (3.87*e* − 62)	1.59*e* − 118 (8.70*e* − 118)	**1.21e − 178 (0.00e + 000)**
*f*_05_	30*e*4	2.96*e* − 15 (1.85*e* − 16)	8.48*e* − 24 (2.31*e* − 24)	6.96*e* − 16 (1.20*e* − 16)	1.47*e* − 045 (7.00*e* − 045)	**1.37e − 061 (5.62e − 061)**
*f*_06_	30*e*4	4.47*e* + 00 (6.09*e* − 01)	2.10*e* + 01 (1.68*e* + 00)	3.77*e* + 01 (3.14*e* + 00)	1.68*e* + 000 (4.05*e* − 001)	**1.01e + 000 (1.62e − 001)**
*f*_07_	30*e*4	0 (0)	0 (0)	0 (0)	0 (0)	**0 (0)**
*f*_08_	30*e*4	3.73*e* − 16 (6.67*e* − 17)	4.65*e* − 91 (7.81*e* − 91)	5.00*e* − 62 (9.38*e* − 62)	7.09*e* − 171 (0)	**5.07e − 241 (0.00e + 000)**
*f*_09_	30*e*4	5.43*e* − 02 (7.03*e* − 03)	6.11*e* − 02 (8.89*e* − 03)	1.14*e* − 01 (1.16*e* − 02)	4.35*e* − 002 (7.69*e* − 003)	**3.11e − 002 (4.60e − 003)**
*f*_10_	30*e*4	3.30*e* + 00 (1.28*e* + 01)	5.04*e* + 01 (5.46*e* + 01)	1.51*e* + 00 (1.34*e* + 00)	**5.27e − 001 (1.18e + 000)**	5.79*e* + 001 (3.71*e* + 001)
*f*_11_	30*e*4	0 (0)	0 (0)	0 (0)	0 (0)	**0 (0)**
*f*_12_	30*e*4	0 (0)	0 (0)	0 (0)	0 (0)	**0 (0)**
*f*_13_	30*e*4	2.47*e* − 04 (1.35*e* − 03)	0 (0)	0 (0)	0 (0)	**0 (0)**
*f*_14_	30*e*4	3.97*e* + 01 (6.47*e* + 01)	3.99*e* − 11 (3.64*e* − 12)	3.56*e* − 11 (2.18*e* − 12)	3.64*e* − 011 (0)	**2.91e − 011 (0)**
*f*_15_	30*e*4	7.31*e* − 14 (5.57*e* − 15)	6.93*e* − 14 (5.00*e* − 15)	1.37*e* − 13 (1.24*e* − 14)	5.93*e* − 014 (7.65*e* − 015)	**2.93e − 014 (5.11e − 015)**
*f*_16_	30*e*4	1.05*e* − 15 (1.21*e* − 16)	7.85*e* − 33 (2.78*e* − 48)	6.19*e* − 31 (3.62*e* − 31)	7.85*e* − 033 (2.78*e* − 048)	**7.85e − 033 (2.78e − 048)**
*f*_17_	30*e*4	1.01*e* − 15 (1.28*e* − 16)	1.35*e* − 32 (5.57*e* − 48)	3.80*e* − 29 (1.87*e* − 29)	1.35*e* − 032 (5.57*e* − 048)	**1.35e − 032 (5.57e − 048)**
*f*_18_	30*e*4	7.34*e* − 07 (1.70*e* − 06)	5.29*e* − 16 (1.25*e* − 15)	8.20*e* − 16 (4.69*e* − 16)	5.42*e* − 047 (2.02*e* − 046)	**3.78e − 066 (9.53e − 066)**
*f*_19_	30*e*4	8.89*e* − 16 (8.73*e* − 17)	1.35*e* − 31 (6.68*e* − 47)	4.08*e* − 30 (2.58*e* − 30)	1.35*e* − 031 (6.68*e* − 047)	**1.35e − 031 (6.68e − 047)**
*f*_20_	30*e*4	9.00*e* − 15 (7.90*e* − 15)	2.42*e* − 14 (8.47*e* − 15)	9.94*e* − 15 (5.68*e* − 15)	0 (0)	**0 (0)**
*f*_21_	30*e*4	4.62*e* − 01 (1.79*e* − 02)	4.61*e* − 01 (1.15*e* − 02)	4.84*e* − 01 (3.62*e* − 03)	4.72*e* − 001 (1.42*e* − 002)	**4.44e − 001 (2.83e − 002)**
*f*_22_	30*e*4	**−7.83e + 01 (4.89e − 14)**	−7.83*e* + 01 (3.71*e* − 11)	−7.83*e* + 01 (2.40*e* − 07)	−7.83*e* + 01 (6.84*e* − 006)	**−7.83e + 01 (6.67e − 014)**
*f*_23_	30*e*4	−1.96*e* + 02 (2.84*e* − 01)	−1.86*e* + 02 (6.01*e* − 01)	−1.74*e* + 02 (9.91*e* − 01)	−1.99*e* + 02 (6.38*e* − 001)	**−1.99e + 002 (7.25e − 002)**
*f*_24_	30*e*4	1.01*e* − 15 (1.23*e* − 16)	0 (0)	5.61*e* − 29 (4.18*e* − 29)	0 (0)	**0 (0)**
*f*_25_	30*e*4	0 (0)	0 (0)	0 (0)	**0 (0)**	2.74*e* − 003 (1.50*e* − 002)
*f*_26_	30*e*4	6.66*e* − 17 (1.08*e* − 16)	0 (0)	0 (0)	0 (0)	**0 (0)**
*f*_27_	30*e*4	7.54*e* − 14 (5.00*e* − 15)	6.90*e* − 14 (4.82*e* − 15)	2.00*e* − 13 (3.07*e* − 14)	**5.97e − 014 (8.52e − 015)**	6.19*e* − 014 (6.26*e* − 015)
*f*_28_	30*e*4	1.24*e* − 05 (5.65*e* − 05)	**1.80e − 16 (1.17e − 16)**	9.71*e* − 16 (5.70*e* − 16)	2.86*e* − 016 (3.41*e* − 016)	4.13*e* − 016 (4.21*e* − 016)

Bold entity refers to one of the best results.

**Table 7:** Comparison of the ranks of the algorithms for the results of 60 and 200 (*f*_22_, *f*_23_) dimensional problems.

No.	Index	GABC	ABCBest1	MABC	ABCVSS	ABCVNS
*f*_01_	Rank	5	3	4	2	1
*f*_02_	Rank	5	3	4	2	1
*f*_03_	Rank	5	3	4	2	1
*f*_04_	Rank	5	3	4	2	1
*f*_05_	Rank	5	3	4	2	1
*f*_06_	Rank	3	4	5	2	1
*f*_07_	Rank	1	1	1	1	1
*f*_08_	Rank	5	3	4	2	1
*f*_09_	Rank	3	4	5	2	1
*f*_10_	Rank	3	4	2	1	5
*f*_11_	Rank	1	1	1	1	1
*f*_12_	Rank	1	1	1	1	1
*f*_13_	Rank	5	1	1	1	1
*f*_14_	Rank	5	4	2	3	1
*f*_15_	Rank	4	3	5	2	1
*f*_16_	Rank	5	1	4	1	1
*f*_17_	Rank	5	1	4	1	1
*f*_18_	Rank	5	3	4	2	1
*f*_19_	Rank	5	1	4	1	1
*f*_20_	Rank	3	5	4	1	1
*f*_21_	Rank	3	2	5	4	1
*f*_22_	Rank	1	3	4	5	2
*f*_23_	Rank	3	4	5	2	1
*f*_24_	Rank	5	1	4	1	1
*f*_25_	Rank	1	1	1	1	5
*f*_26_	Rank	5	1	1	1	1
*f*_27_	Rank	4	3	5	1	2
*f*_28_	Rank	5	1	4	2	3

Average rank		3.786	2.429	3.429	1.750	1.429
Final rank		5	3	4	2	1

**Table 8:** Comparison of ABCVNS and four recent ABC variants on CEC2014 test functions with *D* = 10.

No.	FEs	dABC	qABC	ABCVSS	DFSABC_elite	ABCVNS
F1	10*e*4	2.15*e* + 05 (2.02*e* + 05)	1.46*e* + 05 (1.99*e* + 05)	1.49*e* + 05 (1.17*e* + 05)	8.60*e* + 04 (6.29*e* + 04)	1.22*e* + 05 (9.37*e* + 04)
	Rank	5	3	4	1	2
F2	10*e*4	6.96*e* + 01 (9.15*e* + 01)	6.56*e* + 01 (1.12*e* + 02)	7.25*e* + 01 (1.41*e* + 02)	1.98*e* + 02 (3.98*e* + 02)	4.86*e* + 01 (4.85*e* + 01)
	Rank	3	2	4	5	1
F3	10*e*4	2.85*e* + 02 (2.30*e* + 02)	3.26*e* + 02 (2.89*e* + 02)	3.66*e* + 02 (4.63*e* + 02)	2.52*e* + 02 (3.21*e* + 02)	2.69*e* + 02 (2.69*e* + 02)
	Rank	3	4	5	1	2
F4	10*e*4	2.94*e* − 02 (2.30*e* + 02)	2.34*e* − 02 (3.50*e* − 02)	2.74*e* − 02 (3.03*e* − 02)	3.65*e* − 01 (8.91*e* − 01)	1.03*e* − 01 (1.51*e* − 01)
	Rank	3	1	2	5	4
F5	10*e*4	1.68*e* + 01 (6.69*e* + 00)	1.51*e* + 01 (8.04*e* + 00)	1.60*e* + 01 (6.87*e* + 00)	1.52*e* + 01 (8.00*e* + 00)	1.29*e* + 01 (9.03*e* + 00)
	Rank	5	2	4	3	1
F6	10*e*4	2.18*e* + 00 (7.93*e* − 01)	2.15*e* + 00 (5.92*e* − 01)	2.13*e* + 00 (6.42*e* − 01)	6.88*e* − 01 (3.26*e* − 01)	7.92*e* − 01 (3.65*e* − 01)
	Rank	5	4	2	1	3
F7	10*e*4	1.97*e* − 02 (1.03*e* − 02)	1.16*e* − 02 (9.55*e* − 03)	5.40*e* − 03 (5.36*e* − 03)	4.15*e* − 03 (7.37*e* − 03)	6.41*e* − 03 (7.30*e* − 03)
	Rank	5	4	3	1	2
F8	10*e*4	0.00*e* + 00 (0.00*e* + 00)	0.00*e* + 00 (0.00*e* + 00)	0.00*e* + 00 (0.00*e* + 00)	0.00*e* + 00 (0.00*e* + 00)	0.00*e* + 00 (0.00*e* + 00)
	Rank	1	1	1	1	1
F9	10*e*4	7.80*e* + 00 (2.22*e* + 00)	6.34*e* + 00 (1.84*e* + 00)	5.15*e* + 00 (1.90*e* + 00)	3.67*e* + 00 (1.05*e* + 00)	2.85*e* + 00 (1.26*e* + 00)
	Rank	5	4	3	2	1
F10	10*e*4	1.03*e* − 01 (3.99*e* − 02)	4.34*e* − 02 (5.37*e* − 02)	4.75*e* − 02 (5.19*e* − 02)	3.49*e* − 02 (4.06*e* − 02)	9.24*e* − 02 (7.01*e* − 02)
	Rank	5	2	3	1	4
F11	10*e*4	2.09*e* + 02 (1.32*e* + 02)	1.69*e* + 02 (8.17*e* + 01)	2.00*e* + 02 (9.47*e* + 01)	4.56*e* + 01 (5.21*e* + 01)	8.90*e* + 01 (7.73*e* + 01)
	Rank	5	3	4	1	2
F12	10*e*4	1.31*e* − 01 (2.88*e* − 02)	1.39*e* − 01 (2.52*e* − 02)	1.45*e* − 01 (4.02*e* − 02)	1.22*e* − 01 (3.62*e* − 02)	2.38*e* − 01 (4.76*e* − 02)
	Rank	2	3	4	1	5
F13	10*e*4	1.15*e* − 01 (2.31*e* − 02)	1.20*e* − 01 (2.50*e* − 02)	9.44*e* − 02 (2.42*e* − 02)	8.96*e* − 02 (2.31*e* − 02)	8.00*e* − 02 (1.88*e* − 02)
	Rank	4	5	3	2	1
F14	10*e*4	1.70*e* − 01 (3.10*e* − 02)	1.64*e* − 01 (2.81*e* − 02)	1.58*e* − 01 (3.08*e* − 02)	1.30*e* − 01 (3.36*e* − 02)	1.41*e* − 01 (3.77*e* − 02)
	Rank	5	4	3	1	2
F15	10*e*4	8.66*e* − 01 (2.23*e* − 01)	7.82*e* − 01 (2.18*e* − 01)	6.33*e* − 01 (1.89*e* − 01)	5.65*e* − 01 (1.49*e* − 01)	5.03*e* − 01 (2.28*e* − 01)
	Rank	5	4	3	2	1
F16	10*e*4	1.92*e* + 00 (2.61*e* − 01)	1.90*e* + 00 (3.00*e* − 01)	1.79*e* + 00 (2.71*e* − 01)	1.28*e* + 00 (3.25*e* − 01)	1.54*e* + 00 (4.17*e* − 01)
	Rank	5	4	3	1	2
F17	10*e*4	2.36*e* + 05 (1.49*e* + 02)	1.59*e* + 05 (1.37*e* + 05)	1.63*e* + 05 (1.75*e* + 05)	1.04*e* + 03 (9.99*e* + 02)	1.10*e* + 05 (8.91*e* + 04)
	Rank	5	3	4	1	2
F18	10*e*4	3.67*e* + 02 (2.86*e* + 02)	4.38*e* + 02 (3.76*e* + 02)	7.46*e* + 02 (6.10*e* + 02)	2.56*e* + 03 (2.51*e* + 03)	1.69*e* + 03 (1.67*e* + 03)
	Rank	1	2	3	5	4
F19	10*e*4	3.58*e* − 01 (1.57*e* − 01)	3.21*e* − 01 (1.20*e* − 01)	2.20*e* − 01 (1.19*e* − 01)	1.07*e* − 01 (6.53*e* − 02)	1.03*e* − 01 (6.00*e* − 02)
	Rank	5	4	3	2	1
F20	10*e*4	2.41*e* + 02 (2.88*e* + 02)	1.67*e* + 02 (2.12*e* + 02)	4.78*e* + 02 (6.87*e* + 02)	1.18*e* + 03 (1.51*e* + 03)	7.76*e* + 02 (7.42*e* + 02)
	Rank	2	1	3	5	4
F21	10*e*4	2.50*e* + 04 (2.77*e* + 04)	7.19*e* + 03 (7.43*e* + 03)	1.26*e* + 04 (1.77*e* + 04)	4.29*e* + 03 (4.07*e* + 03)	4.74*e* + 03 (4.10*e* + 03)
	Rank	5	3	4	1	2
F22	10*e*4	1.37*e* + 00 (4.02*e* + 00)	1.59*e* + 00 (3.33*e* + 00)	2.76*e* − 01 (1.49*e* − 01)	2.51*e* − 01 (1.57*e* − 01)	8.82*e* − 01 (3.40*e* + 00)
	Rank	4	5	2	1	3
F23	10*e*4	1.79*e* + 02 (1.23*e* + 02)	1.16*e* + 02 (1.21*e* + 02)	2.72*e* + 02 (1.14*e* + 02)	3.29*e* + 02 (8.68*e* − 04)	3.16*e* + 02 (6.58*e* + 01)
	Rank	2	1	3	5	4
F24	10*e*4	1.21*e* + 02 (4.59*e* + 00)	1.22*e* + 02 (4.65*e* + 00)	1.19*e* + 02 (4.23*e* + 00)	1.12*e* + 02 (2.99*e* + 00)	1.11*e* + 02 (1.64*e* + 00)
	Rank	4	5	3	2	1
F25	10*e*4	1.40*e* + 02 (1.39*e* + 01)	1.32*e* + 02 (7.78*e* + 00)	1.36*e* + 02 (1.05*e* + 01)	1.26*e* + 02 (5.98*e* + 00)	1.26*e* + 02 (5.71*e* + 00)
	Rank	5	3	4	2	1
F26	10*e*4	9.76*e* + 01 (1.27*e* + 01)	1.00*e* + 02 (4.01*e* − 02)	1.00*e* + 02 (4.36*e* − 02)	1.00*e* + 02 (2.95*e* − 02)	1.00*e* + 02 (3.73*e* − 02)
	Rank	5	3	4	1	2
F27	10*e*4	2.12*e* + 01 (7.05*e* + 01)	3.56*e* + 01 (9.63*e* + 01)	8.65*e* + 01 (1.43*e* + 02)	4.27*e* + 01 (1.02*e* + 02)	1.09*e* + 02 (1.49*e* + 02)
	Rank	1	2	4	3	5
F28	10*e*4	3.92*e* + 02 (8.48*e* + 01)	4.01*e* + 02 (2.14*e* + 01)	3.78*e* + 02 (1.58*e* + 01)	3.63*e* + 02 (5.23*e* + 00)	3.63*e* + 02 (9.19*e* + 00)
	Rank	4	5	3	1	2
F29	10*e*4	2.71*e* + 02 (1.93*e* + 01)	2.66*e* + 02 (2.53*e* + 01)	2.68*e* + 02 (2.36*e* + 01)	2.72*e* + 02 (3.00*e* + 01)	2.97*e* + 02 (3.88*e* + 01)
	Rank	3	1	2	4	5
F30	10*e*4	7.47*e* + 02 (1.10*e* + 02)	6.35*e* + 02 (6.60*e* + 01)	6.24*e* + 02 (8.73*e* + 01)	5.86*e* + 02 (8.20*e* + 01)	6.92*e* + 02 (1.75*e* + 02)
	Rank	5	3	2	1	4

Average rank		3.900	3.033	3.167	2.100	2.467
Final rank		5	3	4	1	2

**Table 9:** Comparison among dABC, qABC, DFSABC_elite, and ABCVNS on some test problems with *D* = 30.

No.	maxFEs	dABC	qABC	DFSABC_elite	ABCVNS
*f*_01_	15*e*4	4.21*e* − 13 (3.92*e* − 13)	3.38*e* − 15 (5.42*e* − 15)	4.14*e* − 82 (8.76*e* − 82)	7.26*e* − 125 (3.30*e* − 124)
	Rank	4	3	2	1
*f*_02_	15*e*4	8.76*e* − 08 (1.53*e* − 07)	1.31*e* − 10 (2.21*e* − 10)	5.37*e* − 78 (8.66*e* − 78)	2.77*e* − 122 (1.33*e* − 121)
	Rank	4	3	2	1
*f*_03_	15*e*4	3.75*e* − 14 (1.04*e* − 13)	2.47*e* − 16 (2.43*e* − 16)	2.84*e* − 83 (4.66*e* − 83)	3.33*e* − 126 (1.80*e* − 125)
	Rank	4	3	2	1
*f*_04_	15*e*4	4.09*e* − 26 (1.12*e* − 25)	2.99*e* − 21 (5.31*e* − 21)	2.41*e* − 110 (1.19*e* − 109)	4.76*e* − 162 (2.61*e* − 161)
	Rank	3	4	2	1
*f*_05_	15*e*4	4.26*e* − 08 (1.48*e* − 08)	1.17*e* − 08 (3.31*e* − 09)	2.06*e* − 42 (2.08*e* − 42)	1.38*e* − 065 (3.56*e* − 065)
	Rank	4	3	2	1
*f*_06_	15*e*4	1.02*e* + 00 (5.26*e* − 01)	9.87*e* − 02 (2.30*e* − 02)	5.08*e* − 07 (3.69*e* − 07)	1.03*e* − 002 (2.88*e* − 003)
	Rank	4	3	1	2
*f*_07_	15*e*4	0 (0)	0 (0)	0 (0)	0 (0)
	Rank	1	1	1	1
*f*_8′_	15*e*4	7.18*e* − 66 (5.92*e* − 71)	7.18*e* − 66 (3.50*e* − 72)	7.18*e* − 66 (3.23*e* − 81)	7.18*e* − 66 (8.35*e* − 80)
	Rank	4	3	1	2
*f*_09_	15*e*4	6.19*e* − 02 (1.17*e* − 02)	2.73*e* − 02 (6.83*e* − 03)	1.20*e* − 02 (3.80*e* − 03)	1.30*e* − 002 (3.35*e* − 003)
	Rank	4	3	1	2
*f*_10_	15*e*4	1.38*e* − 01 (1.89*e* − 01)	5.47*e* − 01 (5.00*e* − 01)	3.45*e* + 00 (1.45*e* + 01)	4.32*e* − 001 (1.03*e* + 000)
	Rank	1	3	3	2
*f*_11_	15*e*4	8.16*e* − 13 (1.26*e* − 12)	1.33*e* − 10 (2.15*e* − 10)	0 (0)	0 (0)
	Rank	3	4	1	1
*f*_12_	15*e*4	4.74*e* − 11 (9.50*e* − 11)	5.28*e* − 10 (5.37*e* − 10)	0 (0)	0 (0)
	Rank	3	4	1	1
*f*_13_	15*e*4	4.06*e* − 04 (2.03*e* − 03)	5.47*e* − 12 (2.39*e* − 11)	0 (0)	8.96*e* − 013 (4.89*e* − 012)
	Rank	4	3	1	2
*f*_14_	15*e*4	1.04*e* − 11 (6.27*e* − 12)	4.16*e* − 10 (1.05*e* − 09)	4.37*e* − 13 (1.09*e* − 12)	0 (0)
	Rank	3	4	2	1
*f*_15_	15*e*4	3.83*e* − 07 (1.85*e* − 07)	1.67*e* − 06 (8.22*e* − 07)	3.80*e* − 15 (1.69*e* − 15)	5.15*e* − 015 (4.61*e* − 015)
	Rank	3	4	1	2
*f*_16_	15*e*4	5.56*e* − 14 (6.04*e* − 14)	2.31*e* − 14 (9.81*e* − 14)	1.57*e* − 32 (5.59*e* − 48)	1.57*e* − 032 (5.57*e* − 048)
	Rank	4	3	2	1
*f*_17_	15*e*4	1.94*e* − 13 (2.27*e* − 13)	1.72*e* − 15 (1.86*e* − 15)	1.50*e* − 33 (0)	1.35*e* − 032 (5.57*e* − 048)
	Rank	4	3	1	2
*f*_18_	15*e*4	7.38*e* − 06 (6.48*e* − 06)	8.87*e* − 06 (2.10*e* − 05)	3.10*e* − 40 (1.03*e* − 39)	1.24*e* − 069 (6.33*e* − 069)
	Rank	3	4	2	1
*f*_19_	15*e*4	9.22*e* − 12 (1.02*e* − 11)	2.40*e* − 09 (3.34*e* − 09)	1.35*e* − 31 (2.23*e* − 47)	1.35*e* − 031 (2.23*e* − 47)
	Rank	3	4	1	1
*f*_20_	15*e*4	3.32*e* − 02 (3.58*e* − 02)	1.10*e* − 02 (1.03*e* − 02)	0 (0)	0 (0)
	Rank	4	3	1	1
*f*_22_	15*e*4	−78.332 (2.00*e* − 15)	−78.332 (4.10*e* − 15)	−78.332 (5.02*e* − 15)	−78.332 (4.10*e* − 15)
	Rank	1	2	4	2
*f*_23_	15*e*4	−29.999 (7.48*e* − 04)	−30.000 (1.03*e* − 05)	−30.000 (0)	−29.616 (1.86*e* − 02)
	Rank	3	2	1	4

Average rank		3.227	3.136	1.636	1.500
Final rank		4	3	2	1

*f*_8′_ is a test problem used in [38], and its formula is exp(0.5 *∗* ∑_*i*=1_^*D*^*x*_*i*_).

## Data Availability

The related benchmark problems used to support the findings of this study can be found in this article or the web site https://www.ntu.edu.sg/home/epnsugan/.
